# The Determinacy Problem in Quantum Mechanics

**DOI:** 10.1007/s10701-024-00808-z

**Published:** 2024-11-19

**Authors:** Cristian Mariani

**Affiliations:** Istituto di Studi Filosofici, Lugano (CH), Switzerland

**Keywords:** Collapse of the wavefunction, Measurement problem, Primitive ontology, Quantum indeterminacy, Quantum mechanics, Vagueness, High-dimensional realism

## Abstract

Of the many ways of getting at the core of the weirdnesses in quantum mechanics, there’s one which traces back to Schrödinger’s seminal 1935 paper, and has to do with the apparent *fuzzy* nature of the reality described by the formalism through the wavefunction $\psi$. This issue, which I will be calling the *Determinacy Problem*, is distinct from the standard measurement problem of quantum mechanics, despite Schrödinger himself ends up conflating the two. I will argue that the *Determinacy Problem* is an exquisitely philosophical problem, for as it is standard when facing any phenomenon which appears to have indeterminate or fuzzy characteristics, the solutions available are to either blame the deficiencies of our language, or our lack of knowledge, or to blame the world itself. These three attitudes can already be found in the literature on quantum mechanics, either explicitly or implicitly, and they appear to motivate three very distinct research programs: *high-dimensional realism*, *primitive ontology*, and *quantum indeterminacy*.

In §1 I discuss the roots of what I call the *Determinacy Problem* (DP) in quantum mechanics, and I then motivate why it is useful to provide a rigorous formulation of it. In §2 I formulate the DP as a triad of jointly inconsistent claims. Then, I show that despite its original character, the problem shares structural similarities with the puzzle we face whenever we are to explain any phenomenon that appears to have indeterminate or vague characteristics; namely, we can ask whether the source of the indeterminacy is epistemic, semantic, or ontological. I will then analyse, in §3, each of these three different strategies, and I will show how they relate to existing approaches to the ontology of quantum mechanics. Two major consequences of my analysis will be that: (i) rather than being simply a feature of the theory, *quantum indeterminacy* should be thought of as an ontological framework which is an alternative to forms of *high-dimensional realism* and to the *primitive ontology* program; (ii) the *quantum indeterminacy* framework is strictly connected to *collapse* approaches to quantum mechanics. I will discuss these issue in §4, and will then provide some conclusive remark in §5.

## Schrödinger’s Fuzzy Cat

The problem with quantum mechanics is, for a start, that we don’t really know what the problem is. And this explains why the philosophy of quantum mechanics looks pretty much like a never ending business. As Dennett famously proclaimed [2], there is a sense in which philosophy is that thing that we do when we don’t yet know what the right question is to ask.^1^ There are indeed many possible ways of explaining what is the source of the conundrums in quantum mechanics, and people disagree quite vividly even about how to set the stage for discussion. Among the candidates, there is one which traces back at least to Schrödinger’s [3]. It has to do with the conceptual puzzle arising from the apparent indeterminate nature of reality as it is described by the formalism through the wavefunction $\psi$. Schrödinger asks, quite bluntly, whether we should seriously consider the possibility that the quantum variables themselves, and so the properties of microscopic systems, are inherently *fuzzy* (the German word is *verwaschen*, which translates into ‘washed out’ or ‘blurred’). His own answer is that the *fuzzy model* of reality in unacceptable because such indeterminacy (*Unbestimmtheit*) couldn’t be restricted to the atomic domain and would soon spread, contrary to what we experience, to the world around us:

What is typical in these cases is that an indeterminacy originally restricted to the atomic domain transforms into a rough macroscopic indeterminacy, which can then be resolved by direct observation. This prevents us from naively accepting the “fuzzy model" as a valid representation of reality. There would not be anything unclear or contradictory in the model itself. There is a difference between a shaky or out-of-focus photograph and a snapshot of clouds and fog banks. ([3]: 812)

The passage where Schrödinger explains his reasoning as to why we should reject the *fuzzy model* comes right before, and is certainly one of the most discussed and cited passages of the last century, namely the one containing the infamous cat example. We heard the story many times: a superposition of microscopic states will evolve into a superposed macro-state, unless something happens along the way. In Schrödinger’s own words, this means that if we allow for quantum variables to be inherently fuzzy at the microscopic level, then nothing would prevent them to bring their fuzziness up to the macroscopic scale. Schrödinger here is thinking that (claim *a*) states of superposition represented by $\psi$ could intuitively be interpreted as objectively indeterminate states, but also (claim *b*) that this is problematic due the linear dynamical evolution of quantum mechanics. Electrons may be fuzzy objects, but surely cats aren’t.

It is clear that the argument for thinking that the *fuzzy model* is not viable is based on considerations that have nothing to do, *prima facie*, with the intelligibility of the model (which, to recall Schrödinger’s words, does not have “anything unclear or contradictory” in itself).^2^ Rather, the problem for him has to do with the dynamical evolution of the *fuzzy* states. At this point, we could legitimately wonder why should anyone think that certain features of the dynamics can provide valid reasons to reject an hypothesis about the ontology. After all, it seems at least plausible to suppose that there could be distinct ontologies obeying a dynamics with the same characteristics or, equally, different kinds of dynamical evolutions guiding the very same type of ontology.

There is arguably quite a long history of misunderstanding, especially in the physics literature, about the relationships between two notions that philosophers tend to keep distinct. On the one hand, we speak of determin*ism*, or lack thereof, as a property of laws. On the other hand, we speak of determin*acy*, or lack thereof, as a property of things and stuff. We may very well imagine mixed cases, and this is precisely the reason why philosophers tend to keep the two notions distinct. It is much less clear, especially if we look at the literature on quantum mechanics, whether these notions are always carefully distinguished by physicists. To give but one, notable example, here is David Griffiths’ *Introduction to Quantum Mechanics*—one of the most popular textbooks in the field:

The statistical interpretation [of Born] introduces a kind of *indeterminacy* into quantum mechanics, for even if you know everything the theory has to tell you about the particle (to wit: its wave function), still you cannot predict with certainty the outcome of a simple experiment [...] This indeterminacy has been profoundly disturbing to physicists and philosophers alike, and it is natural to wonder whether it is a fact of nature, or a defect in the theory. ([6]: 4, emphasis in the original)

Notice that here Griffiths is clearly talking about indetermin*ism* (a property of the dynamics), since he refers to the inability to predict with certainty the outcome of experiments. However, shortly thereafter, he imagines to measure the position of a particle and asks “where was the particle just *before* I made the measurement?” This suggests that he then thinks about the indetermin*acy* in the position as a property of the particle itself. As a result, it is not clear what it is exactly that Griffiths thinks physicists find *disturbing*, whether indeterminacy or indeterminism (or perhaps both).^3^

Schrödinger’s reasoning for rejecting the *fuzzy model* may be another example where considerations about the dynamics are mixed up, perhaps erroneously, with considerations about the indeterminacy in the ontology. By this I don’t mean to say that Schrödinger’s reasoning is not sound, for after all it is true that an indeterminate micro-ontology will remain indeterminate at the macro-level if nothing happens in the dynamics. Nonetheless, clearly Schrödinger has two distinct issues in mind here. First, he is thinking about the viability of the *fuzzy model*, an interpretation of the theory which allows for the presence of indeterminacy in the microscopic ontology. In other words, he is asking how we should ontologically interpret the problematic states of superposition. Second, he is clearly gesturing at what will become known as the *measurement problem* (MP) in quantum mechanics, which has to do with whether, how, and why the system collapses into a definite state. While the MP has been the center of a great deal of philosophical discussion on quantum mechanics for almost a century, the former problem, which I will be calling the *Determinacy Problem* (DP), has in general received much less attention and has often been confused with the other problem (pretty much like Schrödinger himself did).

Although the two problems are connected—as Schrödinger’s argument clearly shows—there is a number of good reasons why we should distinguish them. The MP, in most formulations, appears to be a purely scientific problem which has to do with the very consistency of the theory [8]. By itself, however, the MP is silent regarding the ontological implications and commitments. So much so, that often a given solution to the MP may admit of several distinct ontological interpretations. As a consequence, people can agree that certain views are satisfactory *qua* solutions to the MP, but then disagree over whether they are *in general* acceptable based on independent considerations that are the result of broadly ideological, methodological, or even metaphysical intuitions. Moreover, it is far from clear whether just by providing a solution to the MP, we automatically provide a satisfactory explanation of what the theory speaks about (as it is often put: how the world is like if the theory is true). This is precisely what we are witnessing in the recent philosophical debate, where people have started to ask more general questions about the theory, in particular regarding the ontological status of the wavefunction [9], the need for a primitive ontology [10], or the possibility that quantum mechanics entails a deep and pervasive kind of ontological indeterminacy [11].^4^

The main benefit of formulating the *Determinacy Problem* (DP) rigorously is that it will clarify what these various views about the ontology of quantum mechanics may be disagreeing about. I take this to be important for several reasons, but there are two which deserve mentioning. First, one could be tempted to believe that views such as the primitive ontology, the quantum indeterminacy approach, or the wavefunction realism program have little in common. As a matter of fact, one may think that they have so little in common that it is hard to even compare them or judge their merits with respect to one another. This happens arguably because the disagreement enters at the very preliminary and methodological stage, regarding how we think the ontology should be read off from physical theories. As I will show, some of these considerations emerge naturally from the very way in which we can map the solutions to the DP. Second, I suspect that there are more approaches to quantum mechanics than are dreamt in the current philosophical discussion. Interesting and somewhat neglected proposals such as the Modal Hamiltonian interpretation [13], or Rovelli’s Relational view [14], are seldom given adequate attention, and this happens in part because of how philosophers have set up the stage for discussing them. The more these alternative views are incompatible with the standard schemes, the less they will be taken into account in the discussion. In fact, I think this to be true not only for the neglected interpretations, but even for the less standard approaches towards the more discussed ones (I hope it will be clear towards the end of this paper that GRW is a chief example). By giving enough attention to the DP as I am about to formulate it, we may reach a better understanding on how the multiplicity of these views compare to one another.

## The Determinacy Problem Formulated

The *Determinacy Problem* (DP) can be stated rather straightforwardly as an inconsistent triad:

1. $\psi$ does not univocally determine the values of all physical properties.
2. $\psi$ encodes complete information about physical systems.
3. Physical properties instantiate determinate values at all times.

The joint inconsistency of these three claims is self-evident. If (1) $\psi$ does not univocally determine the values of all physical properties, and if (2) $\psi$ encodes complete information about physical systems, then physical properties cannot instantiate determinate values at all times, *contra* (3). If (1) $\psi$ does not univocally determine the values of all physical properties, and if (3) physical properties instantiate determinate values at all times, then $\psi$ cannot encode complete information about physical systems, *contra* (2). Finally, if (2) $\psi$ encodes complete information about physical systems, and if (3) physical properties instantiate determinate values at all times, then $\psi$ must univocally determine the values of all physical properties, *contra* (1).

Each of (1)-(3) appears to be plausible and independently motivated. As for (1), the thought behind it is what motivates Schrödinger’s claim that the “reality corresponding” to $\psi$, in his own words, appears to be *fuzzy*. The reason is simply that since $\psi$ can be in superposed states, and since these states encode observables whose values are not univocally determined, that the reality of $\psi$ is fuzzy just means that the physical properties are not determinate. The justification of (2) is largely* a posteriori*, for it is based on the fact that all the statistical predictions of the theory are expressed by information encoded in $\psi$. If there’s anything more to the ontology than what is expressed by $\psi$, this is not going to make any difference with respect to what we predict. Finally, the justification of (3) is two-folded. First, it appears to be an almost a priori statement the one according to which, if a property is instantiated at all, it is instantiated with a determinate value. Second (and Schrödinger is the first who realizes it), a microscopic system that does not instantiate determinate valued properties will carry such indefiniteness up to to macroscopic scale, unless something happens along the way. This is not however what we experience, since any measurement reveals definite valued properties.^5^

The first, natural comment to what has been said so far, is that the DP appears to emerge from the interpretation of the problematic states of superposition of $\psi$. Suarez [15] goes as far as saying that this issue is *the paradigmatic* one since the advent of quantum physics:

The paradigmatic interpretational question of quantum mechanics is a question about the general interpretation of superposed states: What does it mean - with respect to the property represented by an observable Q - for a quantum system to be in state $\Psi$ that is not an eigenstate of the observable Q? ([15]: 3)

Behind Suarez’s words is the idea that the formalism represents definite-valued properties only when the corresponding observables are in an eigenstate. In other words, the issue he has in mind is strictly connected to the so-called Eigenstate-Eigenvalue Link.^6^ Throughout the years, many have suggested that the correct interpretation of superposition states forces us to accept some kind of indeterminacy.^7^ Still, as much as the claim that $\psi$ involves some indeterminacy may appear intuitive, it is much less clear what *kind* of indeterminacy is supposedly involved. One could indeed accept the presence of some *prima facie* indeterminacy as reflected in $\psi$, but then attempt to explain it away as a merely epistemic limitation. Some others may insist that the indeterminacy arises when we attempt to translate from the mathematical language of the theory—which is perfectly precise and accurate—to the language we ordinarily use. If that’s the case, then there is no indeterminacy left but of a semantic kind. Finally, few would take the indeterminacy at face value as structurally reflecting some property of the world itself.

What is important to realise, is that these three attitudes towards quantum mechanical indeterminacy (semantic, epistemic, ontological) are operative in the ways in which we can overcome the DP. First, one could reject (1) on the basis that the indeterminacy encoded in $\psi$ is due to the fact that the ordinary language fails to translate unambiguously the precise language of the theory. The indeterminacy is therefore purely *semantic*. Second, one can reject (2) convinced that the indeterminacy expressed in $\psi$ is due to our ignorance of the actual, hidden state of the ontology. The indeterminacy would then be considered an *epistemic* matter. Finally, one can reject (3) and insist that the indeterminacy is *ontological*, and it reflects how the world itself is like.

So far, the relationship between the three attitudes towards quantum indeterminacy and the most discussed approaches towards the ontology of the theory has been taken, at best, as a mere uninteresting coincidence. What I am going to suggest in what follows is that there is a correlation between these three attitude and the ways of overcoming the DP, and that these, in turn, roughly correspond to the most discussed ontological approaches to quantum mechanics.^8^ Seen through these lenses, the interpretative issues in quantum mechanics appear in part to be of exquisitely philosophical nature, having to do with vagueness and indeterminacy.

## Three Solutions

I look outside the window and begin staring at a cloud at distance over Lugano lake. If you ask me *where* the cloud is, I can tell you that it is over the lake, next to Monte San Salvatore. If you were to insist and ask about the *exact* position of the cloud, I would tell you that its borders are fuzzy. By saying this, I do not necessarily mean that there is something, the border, which is inherently fuzzy. Perhaps the border is perfectly definite, but I cannot see it clearly. Or perhaps, the word ‘cloud’ I am using to refer to that thing is just a useful short way of referring to a group of molecules that collectively behaves in a certain way. Anytime there is some phenomenon which appears to lack a certain definiteness, I can always take one of these strategies. I can blame the world itself for lacking definiteness, or I can blame my knowledge for lacking certain details, or I can finally blame the language I am using for being imprecise.

Similar examples abound. We may think of the indeterminacy of statements regarding future events, the vagueness in the application of social categories, the undecidability of theorems in logic or mathematics, and so on. We could even think of certain cases where the existence itself of an entity does not have precise conditions for it to be settled. For instance, one year ago my child Vittorio, who’s now 3-month old, was nothing but a bunch of molecules, which makes it hard to tell whether he already existed. Intuitively, one may say, it is indeterminate whether Vittorio existed one year ago. And once again, we can think that such indeterminacy is due to our *ignorance* of the relevant physical facts, and insist that there is a precise instant of time where Vittorio came to existence despite we are unable to assess which one it is. Or else, maybe the word ‘Vittorio’ is a way to refer to a complex physical organism, and although the huge majority of times we use it correctly for our purposes, there are extreme cases where the words fail to refer unambiguously. Finally, one could think that there is nothing in the world which settles the truth of the statement regarding the existence of Vittorio twelve months ago. The world itself leaves this matter unsettled.

The clouds, the mountains, and similarly Vittorio, are macroscopic entities which we assume to be composed of smaller, microscopic stuff. So long as as the indeterminacy is confined to the macroscopic world, many would claim, there really is no deep trouble with the presence of some indeterminacy. Things get more challenging as soon as we take it to be possible that the components of clouds and mountains are themselves inherently indeterminate. This is pretty much what quantum mechanics may suggest, at least according to some people.

Precisely as it happens when facing the phenomena listed above, and which appears to have some indeterminate or vague characteristic, also when asking about the indeterminacy represented in $\psi$ there appear to be three main strategies: to blame our language (semantic indeterminacy), to blame our knowledge (epistemic indeterminacy), or to blame the world (ontological indeterminacy). As I am about to show, these three strategies nicely map to the ways in which we can overcome the *Determinacy Problem*, in that they lead to rejections of (1), (2), or (3), respectively. Moreover, quite interestingly, there is a rough correspondence between these strategies and some of the major research programmes in the discussion on the ontology of quantum mechanics.

### High-dimensional Realism and the Semantic Strategy

The first option is to reject (1), which, to recall, states the following:

(1) $\psi$ does not univocally determine the values of all physical properties.

What are the motivations for endorsing this claim? As said earlier, first and foremost, the idea behind (1) is simply that, just by looking at $\psi$, we won’t be able to always ascribe definite values to all observable properties at the same time. In cases where $\psi$ encodes information about superposed states, we can at best infer certain statistical information about future measurements. So, if $\psi$ is taken to directly represent (or give faithful information about) the values of all physical properties, then we cannot expect $\psi$ to do so univocally in all cases. For, once again, in cases of a superposition state, $\psi$ would represent a *fuzzy* reality, as Schrödinger puts it.

The above conditional claim appears to be true, and yet, some may insist, the real issue is whether or not we should take the antecedent to be true. And to properly assess the truth of the antecedent, many believe that one would first have to clarify what $\psi$ is. As Chen puts it, in a slogan: “Understanding the meaning of quantum mechanics seems to require a good understanding of the meaning of the wave function” [29]. In recent years, the debate on the meaning of $\psi$ has certainly taken centre stage, possibly to the extent that it is now the major point of disagreement in philosophy of quantum mechanics, even more than the measurement problem. In part, this is the case because—some would argue—an understanding of $\psi$ is needed to preliminary set the stage for properly discussing the various interpretations.

Among the many approaches to the question ‘what is $\psi$?’, it is quite straightforward to see that some of them would provide a natural way of rejecting (1). Without getting into much details, a first distinction to be made is between *epistemic* and *ontic* views on $\psi$.^9^ For the former, $\psi$ should be taken to represent the observer’s uncertainty towards the outcome of a given experiment. For the latter, $\psi$ is instead considered as part of the ontology (in a way that needs to be further clarified).^10^ I won’t be focusing too much on the epistemic view here, but it suffices to say that, on this approach, (1) can be rejected on grounds that $\psi$ is not taken to neither determine nor represent *any* physical property whatsoever (since it rather represents the observer’s state of knowledge of the physical systems). Moving to the $\psi$-ontic views, it can be argued that whether or not (1) is accepted depends on what kind of *entity* is $\psi$ supposed to be. There are at least four distinct answers to this question: (i) a field-like object, (ii) a property, (iii) a law, and (iv) a new kind of entity.^11^ Let me discuss these in some detail.

The main distinction, for present purposes, is between (i) on the one side, and (ii)-(iv) on the other. According to (i), $\psi$ is to be considered as a new, field-like kind of object, which is part of our ontology. There are many, interestingly different proposals on how to understand how $\psi$ could be considered a physical field. The main problem is that, contrary to classical fields, $\psi$ appears to live in the high-dimensional configuration space. Yet, on such a view, notice that one could believe that it is *not* strictly speaking the role of $\psi$ to determine the values of physical properties, for in fact, $\psi$ itself is the ontology.^12^ On the contrary, for the three other interpretations (ii-iv above), there is a sense in which to role of $\psi$ is to tell us something about the physical ontology (by being a property of it, or a by giving nomological information about it).

Therefore, endorsing the view that $\psi$ is a field-like object allows us to reject (1) from the DP. To do so, one may insist that $\psi$ is not representing any indeterminacy in the 3d, but rather that it represents a more complex (and determinate) field-ontology which lives in a higher-dimensional space. If that’s the case, then it could be argued that the apparent indeterminacy is purely *semantic*, for it may depend on picking out the 3d as the preferred mode of representation. Somewhat similarly to what David Lewis thought about trying to settle the exact boundaries of a macroscopic object like a mountain, proponents of this approach will insist that it would be foolish to try and settle the precise identity conditions of a microscopic entity like an electron. There are of course crucial differences between the two cases, because the 3d mode of representation isn’t one choice among others, nor does it look anything like a semantic precisification. And moreover, while in the Lewisian case the indeterminacy vanishes as soon as we move to a fully precisified language, in the case at hand the indeterminacy is an unavoidable feature of the 3d description. Nonetheless, strictly speaking this is irrelevant, for if we take the ontology to be given by $\psi$ itself, and this is perfectly determinate, then it should not come as a surprise if our way of referring to it may sometimes be semantically inaccurate or imprecise..^13^ On this view, when we speak of microscopic entities or states of affairs, we are actually just referring, sometimes inaccurately and arbitrarily, to a certain *part* of $\psi$.^14^

Therefore, rejections of (1) are typically coupled with the view that $\psi$ is *not representing* or *determining* the ontology because it *is* itself the ontology. Unsurprisingly, the problem usually associated with these views is that, even granting that $\psi$ is the ontology, we need an explanation of why we seem to be living in 3d space—an explanation, in other words, of how the 3d worlds emerges from $\psi$. Accepting the view that the only indeterminacy in $\psi$ is representational, leads to a representational problem associated with the explanation of the emergence of the macroscopic entities.

To recap, the first strategy to overcome the DP is to reject (1). Although this is not explicitly recognised, the line of research— admittedly quite well spread today—which takes $\psi$ to be a new kind of entity, a field living in high-dimensional space, provides motivations for rejecting (1). By doing so, the indeterminacy is resolved as a purely semantic phenomenon.

### Primitive Ontology and the Epistemic Strategy

The second strategy is to reject (2):

(2) $\psi$ encodes complete information about physical systems.

The straightforward way to do so is to deny that the information encoded in $\psi$ is complete. If that’s the case, then one could argue that whatever more there is, this is going to provide a fully determinate characterisation of the ontology. This move comes at a cost, however, for there seem to be very good reasons to believe that $\psi$ encodes everything there is to know about physical objects. In every experimental context, the information about future measurements is given a certain statistical weight by properly looking at $\psi$. Even if there were to be something more, which was not represented by $\psi$ in any sensible way, that something would be admittedly irrelevant when it comes to making predictions. Of course, one could say, not everything physics needs to postulate must be useful for predictive purposes. Perhaps, in some cases, positing additional ontology is required precisely in order to provide a clear and more intuitive picture, and to explain reductively the behaviour of macroscopic objects in terms of the behavior of the microscopic ones.

Leaving the details aside, similar considerations are made by people who argue in favor of the so-called *Primitive Ontology* program [10]. This approach suggests that we should accept the view that *there is more* to the ontology than what is described by $\psi$. Whatever more is added, it is going to be determinate almost by definition.^15^ Therefore, any indeterminacy represented by $\psi$ will be of the *epistemic* kind; it just is lack of knowledge as regards to the actual state of the (determinate) ontology.^16^ This second approach adds something to the ontology to solve what appears to be an ontological problem. For proponents of this view, the main concern we must address when providing an ontology for quantum mechanics is that of giving a picture that is as *intuitive*, as *determinate*, and as *classical* as possible. In a very precise way, this approach is designed in order to give an alternative to the *high-dimensional realism* and to the *indeterminacy* proposals.

There is a lot that makes us feel comfortable about this view. For one, the resulting picture is one that is engineered to resemble to our classical explanatory schemes. Furthermore, accepting that quantum indeterminacy is just an epistemic phenomenon seems to be, all else being equal, the most obvious thing to do. There are however drawbacks with this view too. As mentioned, one issue has to with how we can justify the appeal to an in principle hidden ontology. If the main task we demand the primitive ontology to accomplish is to recover classicality, then someone would consider *ad hoc* the move so long as quantum mechanics by itself provides lots of reasons to revise our classical notions. Moreover, there is another crucial concern that one could have when looking in more details at the PO program. Recall from §3.2 that there are various ways for explaining what’s the ontological status of $\psi$, and among those is the idea that $\psi$ is a law of nature (albeit of a very peculiar nature^17^). Arguably, the primitive ontology view is connected to the acceptance of this thesis.^18^ As much as this view is not problematic *per se*, it surely does entail that $\psi$ being a law does a lot of explanatory job.

PO approach can be interpreted as a way to reject (2) from the DP, and thus as a way to insist that quantum indeterminacy is merely *epistemic*. The superposed states are not ‘problematic’ anymore, since they simply represent our lack of knowledge as regards to the actual state of the primitive, determinate ontology.

### Quantum Indeterminacy and the Ontological Strategy

The third and final strategy to overcome the DP is to reject (3), which to recall states that:

(3) Physical properties instantiate determinate values at all times.

Schrödinger’s *fuzzy model* is, to the best of my knowledge, the first explicit suggestion in this direction. By rejecting (3) we thereby take at face value the indeterminacy encoded in $\psi$, and accept that it is of the ontological kind. Once we take $\psi$ to be a complete and faithful representation of reality, it follows that nature is itself indeterminate in some respect. This approach, as mentioned, leads to two main problems.

First, the motivation behind (3) is in some sense purely conceptual, for it has to do with how we could even make sense of the thesis that the world is itself indeterminate. This explains why some take Schrödinger’s arguments as a *reductio* of the model ([4, 5]). It may even look as *a priori* the statement according to which if an object instantiates a physical property, it does so with a precise and definite value. This is surely a problem that many would consider a crucial one,^19^ and yet it is unclear how much it should really stop us from developing this possibility. In one way or another, quantum mechanics will require a huge departure from our classical intuitions. It is possible that the source of the departure, and so the difference between classical and quantum physics, is precisely the existence of ontological indeterminacy.^20^

The second problem with rejecting (3) is the one correctly individuated by Schrödinger. Even if we were to make sense of the strange hypothesis that the world is indeterminate (that the reality corresponding to $\psi$ is *fuzzy*), we would still need to explain how it happens that the indeterminacy gets restricted to the microscopic world. This problem is one which pertains to the dynamical evolution of the indeterminate states, and it is strictly related to the idea of the *collapse* of $\psi$. It is at this stage that *determinacy problem* interacts more clearly with the solutions to the *measurement problem*. The idea that the reality corresponding to $\psi$ is inherently indeterminate demands an explanation of why there are no macroscopic superposition states, such as those of a cat being both dead and alive at the same time. Thus, the possibility that something—be that a conscious observer or the dynamics itself—makes $\psi$ collapse to a definite state, appear to be naturally linked to the rejection of (3) and to the acceptance of ontological indeterminacy. Also notice that, if that’s the case, then it should not come as a surprise that Schrödinger rejected his *fuzzy model*. After all, back in 1935, there were no clear proposals on how to properly explain *collapse*. Today the situation is radically different, as there are plenty of proposals on how to understand collapse either as a physical mechanism (as for the GRW model [47]), or anyway as being independent from the measurements and observers (as in Rovelli’s proposal [14]). Perhaps, if Schrödinger were to know about these advancements in our understanding of collapse, he himself would reconsider the viability of the *fuzzy model*.

To sum up, the third major strategy to overcome the DP is to deny (3). This arguably leads to the acceptance of the view that quantum indeterminacy is ontological. As I have shown, the rejection of (3) lead to the dynamical problem connected to the explanation of how the indeterminacy is restricted to the microscopic domain. As one would expect, a problem in the dynamics asks for a solution in the dynamics. This is, in a nutshell, precisely what the collapse of $\psi$ is supposed to do.

## Indeterminacy and Collapse

One of the conclusions to be drawn from the discussion of the DP is that the approach I have been calling *quantum indeterminacy* should be regarded as an alternative view both to forms of *high-dimensional realism* and to the *primitive ontology* program. These three families of views attempt to provide distinct answers to the DP, and as a consequence they compete in the task of providing an ontological framework for quantum mechanics.^21^ If this is correct, then I think we should partially reconsider the idea, expressed by several people,^22^ that quantum indeterminacy is a feature of the theory among others. As it should be clear by now, the way I see it is rather different; *quantum indeterminacy* should be thought of as a novel and alternative framework on the ontology of quantum mechanics.

This is I believe an insightful conclusion for many reasons, but mostly because it provides grounds for considering more carefully the interplay between *ontological indeterminacy* and *collapse*. So far, there have been discussions regarding the possibility that quantum indeterminacy, considered as ontological, may affect interpretations where collapse is absent.^23^ In my opinion, this has lead to some confusion regarding what exactly the notion is supposed to be useful for, thereby also generating skepticism towards the whole program. After all, it is true that *if* quantum indeterminacy were to exist in every interpretation, and *if* it were possibly a feature of approaches so different from one another such as the Bohmian and the Everettian, then surely one would have good reasons to be skeptical that there is anything deep going on with it. The obvious consequence of this, unfortunately, would be that we may miss the opportunity to appreciate how useful the notion could be—and, as matter of fact, *already* is—in contexts where it is more natural to be applied, namely in *collapse* interpretations.

Whatever you think quantum collapse is, there is something which is arguably essential to it. When we say that a microscopic system collapses, we want to indicate that there has been some change in its state, and that the change is such that the post-collapse state is a determinate one. I take this to be true for every possible understanding of collapse: we first have some indefinite state, and then, after collapse has happened, we get a definite one. ‘Collapse’ is, in a quite precise sense, the name we give to whatever process *explains* how we get determinate states from indeterminate ones.^24^ Therefore, my claim is that *quantum indeterminacy*—as a framework that is meant to be an alternative to the major existing views on quantum mechanics—should be considered as a way of understanding the ontology of collapse views of quantum mechanics. When interpreted in this way, this framework appears to be quite efficient and useful, as a quick overview of the literature can show.

Different approaches to the meaning of the collapse mechanism can be classified as follows:

1. *Primitive Collapse*. Von Neumann [53] famously introduced the notion of collapse, which he considered as an explained primitive. Up until today, this understanding is widespread about physicists, and it is a crucial component of what we call the *standard interpretation*. On this view, collapse happens whenever we make a measurement, and act as a way to break the linear evolution.
2. *Spontaneous (objective) Collapse*. This approach has been defended by several physicists, and most notably by Ghirardi and collaborators [47]. In this view, collapse is an objective mechanism which happens spontaneously and it is reflected in the dynamical evolution. This understanding of collapse is what characterises a whole family of realist interpretations.
3. *Democratic Collapse*. This approach has been defended by Rovelli within its relational interpretation [14]. Rovelli is explicit that collapse should be made *democratic*, by which he means that *any* kind of interaction between *any* system is enough to collapse their state.

For each of the above approaches, there has been already several applications of the notion of *quantum indeterminacy*.

We find the claim, more or less explicit, that quantum indeterminacy can provide a better understanding of the standard interpretation in [24, 48, 49, 54], and [55]. These authors seem to share the idea that, despite the problems that a *primitive* understanding of collapse may have, indeterminacy can at least provide an ontological framework for this interpretation.

As for the *spontaneous and objective collapse* approach, it has been argued in [56] and [43] that the notion of *quantum indeterminacy* allows us to overcome some crucial problems of this interpretation (most notably, the *tails problem*). Although people have argued that the spontaneous collapse models should be understood either in terms of a *primitive ontology* as in [10], or else within the context of *high-dimensional realism* as in [31], there are doubts that the indeterminacy can be fully resolved in both these views, as extensively argued in [43]. Furthermore, there are good reasons to think that the proponents of the view themselves take the model as suggesting the existence of quantum indeterminacy. Here are Ghirardi and Bassi, who interestingly also make an analogy between their own understanding of the ontology of the model in terms of a Mass Density [57] and Schrödinger’s *fuzzy model* we met in §2:

This analysis shows that one can consider at all levels (the micro and the macroscopic ones) the field m(x,t) as accounting for ‘what is out there’, as originally suggested by Schrödinger with his realistic interpretation of the square of the wave function of a particle as representing the ‘fuzzy’ character of the mass (or charge) of the particle. Obviously, within standard quantum mechanics such a position cannot be maintained [but] the picture is radically different when one takes into account the new dynamics. [58]

This comment from Ghirardi and Bassi perfectly closes the circle. Their idea here is clearly that, once collapse is understood properly as a physical mechanism, we no longer have to face the problem Schrödinger felt the *fuzzy model* would have. Therefore, at this point it is quite natural to recover the model and accept ontological indeterminacy as represented by the “fuzzy character of mass”.

Rovelli’s relational quantum mechanics has been given an interpretation within the quantum indeterminacy frameworks in [59]. Once the collapse process is understood, in Rovelli’s terms, as happening at every micro-physical interaction, it becomes legitimate to ask what we should think about *non-interacting systems* (systems before collapse). The suggestion in [59] is that these can be interpreted as indeterminate states.^25^ This is a quite natural path to take, for once again it is based on the intuition that collapse only makes sense if it is meant to explain the becoming determinate of previously indeterminate states.

All of this shows that if we take *quantum indeterminacy* to be an alternative framework on the ontology of quantum mechanics, this can potentially help in understanding the ontology of *collapse* approaches to the theory. This is important also because it allows us to see the similarities between the various approaches to collapse (standard, spontaneous, democratic), and to appreciate their differences with respect to approaches that deny collapse (Everettian, Bohmian, and so on). This also helps us evaluating the merits and demerits of the collapse views without attempting to force them into frameworks (such as the *high-dimensional realism* and the *primitive ontology*) that were clearly *not* designed for them.

What I have in mind when I say the *quantum indeterminacy framework* is still something to be fully developed in details. There are several interesting issues that have to be addressed, for instance regarding the ontological meaning of $\psi$ or the consequences of *non-locality*. However, there are already very good reasons to think that, on the one hand, *quantum indeterminacy* can help understanding the ontology of collapse, and on the other, *collapse* should be taken as a crucial starting point when applying the notion of *quantum indeterminacy*.

## Conclusive Remarks

I take it that the main lesson to be drawn from having properly classified the solutions to the DP, is that we now have a possible explanation of the source of disagreement among the major ontological views on quantum mechanics (namely *high-dimensional realism*, *primitive ontology*, and *quantum indeterminacy*). There are obviously several distinct motivations that pull us towards one view of the other. These have to do, in some cases, with the internal consistency and viability of the proposals themselves. For example, we have seen how the *ontological* approach towards quantum indeterminacy requires some further dynamical explanation regarding how the collapse of $\psi$ works. In other cases, the motivations for choosing one over the other solutions may have to do with more general considerations, perhaps methodological. But all of this, as I have argued, can be taken to derive from a preliminary attitude towards the understanding of the phenomenon of quantum indeterminacy as being *semantic*, *epistemic*, or *ontological*. The problem of quantum mechanics, seen in this way, could not be more philosophical, since it reminds us in some insightful ways the problem of vagueness [61, 62].

## Data Availability

No datasets were generated or analysed during the current study.

## Footnotes

1 I owe a profound debt to Thomas Sattig for stimulating discussions on this.

2 We are often told a different story, according to which Schrödinger’s argument is meant as a *reductio* of the fuzzy model *and* an argument for claiming that quantum mechanics is incomplete (e.g. Norsen ([4]: 74) and Bricmont ([5]: 39)). Such a reconstruction does not do justice to Schrödinger, who is clearly saying that the fuzzy model is not contradictory in itself. Norsen and Bricmont, among others, seem to focus only on Schrödinger’s claim *b* made above, while they fail to recognise claim *a*.

3 The first reactions to Bohm’s proposal in the early ’50 s show how hard it is to get a clear understanding of what physicists really find disturbing. As recently shown in [7], Bohm was mostly concerned with providing a clear ontological picture for quantum theory. Therefore, one could say, he was mainly concerned with avoiding indetermin*acy* However, many people misunderstood him as attempting to argue against the indetermin*ism* of quantum mechanics.

4 Allori explicitly argued in a recent paper [12] that the MP as a problem is independent from the problem of how to understand the ontology of the theory from the perspective of scientific realist. She calls this latter issue the *realism problem.*

5 It is worth noticing that there is an asymmetry between claims (1)-(2) on the one hand, and claim (3) on the other, in that the latter is not a claim explicitly about quantum mechanics. As a consequence, one could have reasons that are independent from quantum mechanics to reject claim (3), especially in cases of emergent or derivative properties. Therefore, one would be led to reject claim (3) more easily, since it may have to go anyway. As sympathetic as I could be to this attitude, I take it that most people are naturally led to assume that the definiteness of values for any given property is an almost *a priori* principle. And crucially, this is generally considered to be case both for fundamental and for derivative or emergent properties. Of course, if you think otherwise, you will most certainly welcome rejections of (3) also in the case I’m discussing here.

6 The importance of this link has been put into severe questioning by Wallace [16]. Gilton [17] has however provided good reasons to dismiss Wallace’s concerns. The EEL has been at the core of the discussion on quantum indeterminacy (see [11] for a review, and [18] for an extensive analysis).

7 Examples include Salmon [19], Bub [20], Lowe [21], Hawley [22], Busch [23], and Calosi and Wilson [24, 25]. In recent years, there have been systematic attempts in understanding quantum indeterminacy as an ontological phenomenon. For references, see [11] and [26]. For criticism, see [27] and [28].

8 I say “correlation” and “roughly correspond” because I do not mean to suggest any straightforward link, nor anything like a one-to-one mapping, between attitudes towards indeterminacy and existing programs in quantum ontology. In some cases, as it will be clear, the connection is stronger than in others. What really matters, I believe, is that once we realise that there is some such correlation, we begin asking more detailed questions about specific cases.

9 Some [29] would distinguish between the *epistemic* and *instrumentalist* views. For everything that follows this distinction will be irrelevant, and everything I say about the former will also apply to the latter.

10 In their highly influential paper, Pusey et al. [30] show that some $\psi$-epistemic views can be rejected on empirical grounds.

11 (i) is introduced by Albert [31], and then defended in [32–34]. An interestingly different view, but still to be considered within option (i), was defended by Hubert and Romano [35]. (ii) is defended, in different ways, by Monton [36, 37] and Wallace and Timpson [38]. (iii) is introduced [39] and defended by many proponents of the primitive ontology approach. (iv) is defended by Maudlin in [40]. For a discussion and comparison, see [29].

12 Some would go as far as claiming that it is the *fundamental* ontology [41], but this issue doesn’t matter for our purposes; so long as $\psi$ is viewed as an object, no matter whether fundamental or not, we have a way to reject (1) from the DP. Of course, all of this will depend significantly on what one believes the relationships between $\psi$ and the derivative ontology is.

13 To be clear, this is just one possible understanding of what is going on here, and I do not mean to argue that it is the most natural one. Of course, there are various other views on which, on the contrary, we don’t have any semantic indeterminacy either (e.g. arguably, on views such as state-space realism [38]). Moreover, one could believe that the presence of semantic indeterminacy, by itself, doesn’t exclude the existence of emergent ontological indeterminacy. Once again though, it is unclear how much such a view would be appealing to those who believe that ontological indeterminacy isn’t consistent, independently of whether it appears at the fundamental or at the derivative level.

14 Although the intuition behind this is certainly understandable, there could be doubts about how to make sense of mereological vocabulary in this context—especially given considerations regarding *entanglement* I am not going to discuss this further, though see [42] for discussion.

15 Though see [43] for a critique that the determinacy is indeed an essential requirement for this view.

16 I set aside an important caveat, for it would lead me too far to be properly discussed, and yet I think it deserves to be mentioned. Strictly speaking, from what I said so far nothing would prevent us from having indeterminacy in properties that are not fundamental. Lewis [44] makes the example of *spin* in Bohmian mechanics. To cover such cases, at the very least, we would have to require that the only physically relevant properties are the fundamental ones.

17 See [29] for discussion.

18 Though it’s not always the case, as for example in [35] and [40].

19 For instance, both Glick [28] and Menon [45] argue against the existence of quantum indeterminacy based on broadly methodological considerations.

20 Moreover, it must be realised that the criticism should not be one of *inconsistency* The idea that world is itself indeterminate may be hard to conceptualise, but it is far from being incoherent. This has been shown by many in the past two decades, and it is surely an important advancement in our philosophical understanding of the notion of indeterminacy. See [46] for a recent review of the topic.

21 I should be clear that I *do not* consider these views to give an exhaustive list of all possible approaches. I do think, however, that other views will agree more or less with these three depending on how they attempt to solve the DP. All else being equal, one would arguably prefer to reject just one of the above claims, rather than two or three. Of course though, this is not mandatory, precisely because isn’t not often the case that all else is equal. There are, as a consequence, some quite important views on which more than one claim is rejected. My aim here is not to argue that these view shouldn’t be considered, nor that they deserve less attention. My goal was rather to show the more simple cases, so as to better deliver what is the general structure of the DP. I thank an anonymous reviewer of this journal for inviting me to say more here.

22 This is arguably the original idea expressed in [48, 49] and [24].

23 Examples include [44, 50–52].

24 Contrary to what happens, for instance, with decoherence, where there is arguably, strictly speaking, no *explanation* of the transition from the previous to the later states. While I do not mean this comment to be exhaustive regarding the intricate connection between *collapse* and *decoherence*, or about their analogies and disanalogies, I think most people would agree on my previous claim that the two notions are distinct when it comes to providing an explanation of the transition from indeterminacy to determinacy. I thank an anonymous reviewer for pushing on this.

25 See [60] for an alternative view.
